# Gut microbiota in early and established stages of rheumatoid arthritis: from pathogenesis to promising prevention and treatment

**DOI:** 10.1080/07853890.2026.2613484

**Published:** 2026-01-23

**Authors:** Shuanglan Chen, Minh Hung Hoang, Dongsen Hu, Qingman He, Yongxiang Gao

**Affiliations:** a Department of Rheumatology and Immunology, Hospital of Chengdu University of Traditional Chinese Medicine, Chengdu, China; b Sichuan Jinxin Xi’nan Women’s and Children’s Hospital Co., Ltd, Chengdu, China; c Quang Binh Medical College, Quang Binh, Vietnam

**Keywords:** Early stages of rheumatoid arthritis, established stages of rheumatoid arthritis, gut microbiota, pathogenesis, treatment

## Abstract

**Background**: Rheumatoid arthritis (RA) is a chronic autoimmune inflammatory disease whose etiology is not fully understood. Before overt disease, genetically prone individuals may undergo a lengthy pre-clinical phase during which loss of tolerance triggers autoantibody emergence. The gut–joint axis was proposed decades ago, and mounting evidence now suggests that alterations in the gut microbiome may matter most in early, rather than established, RA.**Results**: By modulating intestinal permeability, mucosal and systemic immunity, genetic-risk pathways, molecular mimicry, and microbial metabolites, the gut microbiota plays a pivotal role in the onset and progression of RA. The window of opportunity for RA therapy may lie before joint inflammation becomes evident, and microbiota-targeted interventions are emerging. Probiotics, dietary interventions, and natural compounds hold promise as potential strategies for both RA prevention and adjunctive therapy after onset.**Conclusions**: This review highlights the gut microbiota not merely as a modulator of established RA, but – more critically – as a driver of early disease pathogenesis and a promising therapeutic target, thereby providing new insights for managing pre-clinical RA and refining treatment of established RA.

## 1. Introduction

Rheumatoid arthritis (RA) is a chronic autoimmune disorder marked by persistent synovial inflammation and progressive joint destruction, systemic involvement, and the generation of autoantibodies such as anticitrullinated protein antibodies (ACPAs), rheumatoid factor (RF), and anti-carbamylated peptide antibodies. Although its precise pathogenesis remains unresolved, early hypotheses proposed that certain genetic variants might drive joint inflammation and disease development [1]. Subsequent twin studies, however, revealed high heritability but limited concordance for RA [2,3] indicating that genetic predisposition alone rarely initiates the condition. Environmental cues are now thought to act as critical triggers in individuals carrying susceptible genetic backgrounds. The gut, which forms a major interface between the internal milieu and the external environment, hosts a complex microbial ecosystem that has co-evolved with the human host. As an influential environmental factor, the gut microbiota plays a pivotal role in shaping the onset and trajectory of autoimmune disorders.

Genetically susceptible individuals may enter a prolonged preclinical stage long before arthritis becomes clinically or histologically apparent, during which environmental exposures interact with inherited risk to initiate and drive disease progression [1]. Mucosal microbial dysbiosis closely correlates with localized autoimmune activity, and extensive evidence supports the concept that RA originates at mucosal surfaces, including the oral cavity and the intestinal tract. The intestine contains the body’s largest reservoir of innate and adaptive immune cells and is widely regarded as its most substantial immune organ [4]. Gut microorganisms shape host immunity and influence RA pathogenesis through a wide array of mechanisms [5]. More and more evidence supports the ‘gut–joint axis’ hypothesis and emphasizes the significance of the microbiota. Alterations in the gut microbiota community during the earliest stages of RA differ markedly from those observed once the disease becomes established. Attributing pathogenic significance to late-stage microbial signatures risks obscuring stage-specific features that may be relevant for disease initiation. Individuals in the preclinical phase (PreRA) often display gut microbiota compositions resembling those of patients with new-onset untreated RA (NORA), and the microbial profiles in both groups appear largely insulated from the confounding effects of systemic inflammation or pharmacologic therapy. Consequently, this review focuses on human PreRA and NORA cohorts, alongside transgenic mouse lines that spontaneously develop arthritis and rodent models sampled during the immune-priming phase, to elucidate how perturbations in gut microbiota may precipitate RA onset. Individuals with established RA who do not meet NORA criteria and animals with experimentally induced arthritis represent the established-disease and are considered separately from the early-phase populations examined here.

**Figure 1.**
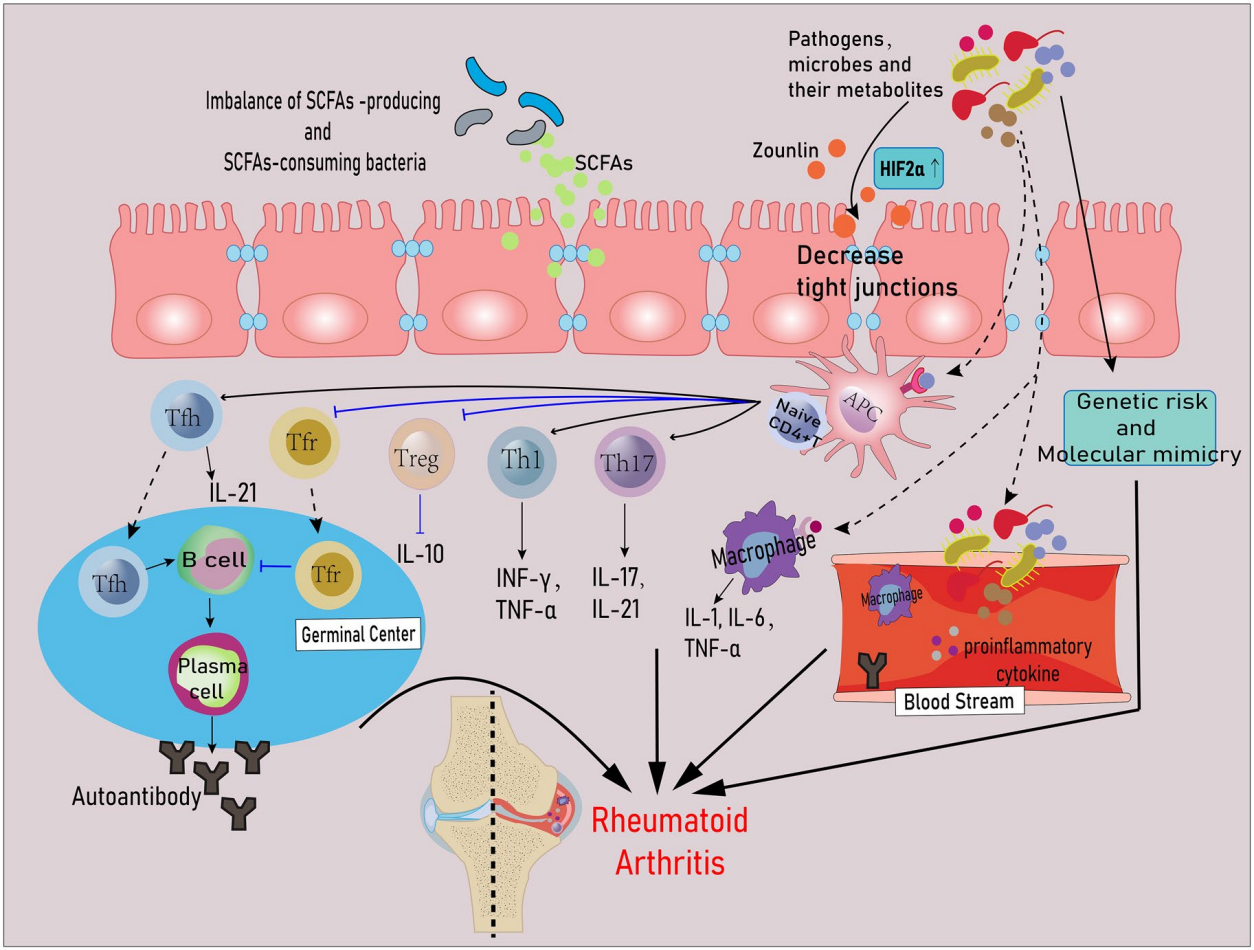
Mechanisms of gut microbiota in the etiology of RA. During the onset and progression of RA, alterations in the gut microbiota may mediate the disruption of the intestinal mucosal barrier, and increased levels of zonulin can also enhance intestinal permeability. Bacteria or their components pass through the interstitial spaces of the intestinal epithelium into the secondary lymphoid organs or the bloodstream and are transported to the joints. Naive CD4+ T cells rapidly differentiate into distinct cell subpopulations and produce various effector cytokines upon receipt of microbe-derived antigens presented by antigen-presenting cells. Tfh cells activate B cells at GC to promote their differentiation into plasma cells and production of autoantibodies, whereas Tfr cells inhibit the differentiation of B cells into plasma cells. In addition, microbe-derived antigens directly stimulate the production of pro-inflammatory cytokines by macrophages. Thus, gut microbes are involved in the pathogenesis of RA by altering intestinal permeability, modulating immune responses, influencing genetic risk and molecular mimetic pathways.

This review outlines stage-specific microbial alterations across RA development, delineates the diverse mechanisms through which the microbiota contributes to disease initiation and progression (Figure 1), and highlights the therapeutic promise of microbiota-targeted interventions for RA prevention and management. The overarching aim is to refine understanding of how environmental factors shape RA pathogenesis and to identify strategies that may avert disease emergence or improve outcomes once RA becomes clinically evident.

## 2. Alterations in the intestinal flora in RA

Existing studies have yielded important insights into the relationship between the gut microbiota and both early-stage and established RA (Table 1). One recurrent signature in the gut microbiota of PreRA and NORA individuals is an expansion of *Prevotella –* particularly *Prevotella copri* (*P. copri*) *–* accompanied by reduced abundance of *Bacteroides* and the loss of several beneficial taxa [6,7]. In contrast, patients with established RA who have undergone treatment and achieved lower disease activity typically display *P. copri* levels comparable to those of healthy controls [6,8]. Notably, clinical cohorts in China have not reported an increase in *Prevotella* during either the early or established stages of RA, diverging from findings in the United States, Japan, and other regions. This divergence highlights the considerable geographic, regional, and ethnic heterogeneity of RA-associated gut microbial profiles.

**Table 1.** Summary of studies on gut microbiota dysbiosis in early and established stages of RA individuals and mouse models.

Subjects	Country	Type of study	Method	Gut microbiota in RA	Key findings	Reference
PreRA(53) VS Healthy control (HC)(38)	China	Human study (case control study)	16S rRNA	**Genus:** *Lactobacillus*, *Raoultibacter*, *Eubacterium_brachy_group*, *Enorma, Holdemania*↑; *Ruminococcus_2*, *Pseudomonas*, *Ruminiclostridium_5*, *Coprococcus_2*, *Ruminococcaceae_UCG-009*, *Chryseobacterium↓*	Bacterial community richness and diversity were significantly reduced. Kyoto Encyclopedia of Genes and Genomes (KEGG) pathway enrichment analysis reveals amino acid and lipid metabolism enrichment. PreRA ecal microbiota transplantation (FMT) mice show increased intestinal permeability and Th17 cell activation and enhanced collagen-induced arthritis (CIA) severity.	[19]
NORA(26) VS HC(26)	China	Human study (case control study)	16S rRNA (regions V3-V4)	**Genus:** *Klebsiella*, *Escherichia*, *Eisenbergiella*, *Flavobacterium*↑; *Clostridiales,* *Fusicatenibacter*, *Megamonas*, *Enterococcus*↓	Alpha diversity of the intestinal microbiome was reduced. Function prediction analysis demonstrated that the biosynthesis pathways of amino acids, such as L-arginine and aromatic amino acids, were depleted in the NORA group.	[8]
NORA(15) VS HC(15)	China	Human study (case control study)	16S rRNA	**Genus:** *Lactobacillus*↑; **Species:** *L. salivarius*, *L. iners*, *L. ruminis*, *L. mucosae*↑	Increased diversity of fecal lactobacilli and bacterial varieties in NORA patients.	[148]
①NORA(25) VS HC(22) ②RA(37) VS HC(13)	China	Human study (case control study)	16S rDNA	**①Genus:** *Parabacteroides*↓ **Species:** *Parabacteroides distasonis*↓ **②Phylum:** *Bacteroidetes*↓; *Verrucomicrobia*↑ **Genus:** *Bifidobacterium, Agathobacter, Akkermansia, Ruminococcus, Veillonella*↑; *Erysipelothichaceae, Roseburi, Dorea, Erysipelatoclostridium, Faecalibacteriu, Bacteroides, Subdoligranulum, Alistipes, Coprococcus, Parabacteroides, Blautia*↓ **Species:** *Parabacteroides distasonis*↓	*Parabacteroides* distasonis abundance in RA and NORA patients was negatively correlated with DAS28. Oral administration of *Parabacteroides* distasonis in CIA mice improved joint inflammation by correcting Th17/Treg cell imbalances in the spleen and intestine.	[9]
RA(17) VS HC(14)	Japan	Human study (case control study)	16S rRNA (V5-V6)	**Genus:** *Prevotella*↑; *Bacteroides*↓ **Species:** *P. copri*↑	SKG mice with NORA microbiota had an increase in gut Th17 cells and developed more severe arthritis than SKG mice with HC microbiota. SKG mouse spleen naive CD4+ T cells co-cultured with P. copri stimulated DC produce high levels of IL-17 in response to RA autoantigen RPL23A.	[40]
①NORA(44) VS HC(28) ②RA(26) VS HC(28)	America	Human study (case control study)	16S rRNA (regions V1–V2, 454 platform)	①**Family:** *Prevotellacea*e↑; *Lachnospiraceae*↓ **Genus:** *Prevotella*↑; *Clostridia*, *Bacteroides*↓ **Species**: *P. copri*↑ ②**Family:** *Bacteroidaceae*↑ **Genus:** *Bacteroides*↑	Analysis of enzymatic functions in the *Prevotella*-dominated metagenome reveals a significant decrease in purine metabolic pathways, including tetrahydrofolate biosynthesis. Patients in the NORA cohort showed a significant inverse correlation between *P. copri* relative abundance and presence of SE alleles. Colonization of mice revealed the ability of *P. copri* to dominate the intestinal microbiota and resulted in an increased sensitivity to chemically induced colitis.	[6]
RA(40) VS HC(32)	America	Human study (case control study)	16S rRNA (regions V3-V5)	**Phylum:** *Actinobacteria*, *Bacteroidetes*↑ **Genus:** *Collinsella*, *Eggerthella*, *Actinomyces*↑; Faecalibacterium↓ **Species**: *Collinsella. Aerofacien*s, *Eggerthella. lenta*↑	Reduced gut microbial diversity in RA patients, which correlates with disease duration and autoantibody levels. *Collinsella* increases intestinal permeability and disease severity in CIA mice.	[12]
PreRA(83) VS HC(50)	Germany	Human study (case control study)	16S rRNA (regions V4)	**Family:** *Prevotellaceae*↑ **Genus:** *Prevotella*↑ **Species:** *P. copri*↑	Differences in α and β diversity between the two groups were not significant.	[7]
DBA/1J Mice before CII-enhanced immunization VS Naïve DBA1/J	/	Animal study	16S rRNA (regions V4)	**Phylum:** *Firmicutes*, *Proteobacteria*↑; *Bacteroidetes*↓ **Family:** *Lachnospiraceae*, *Ruminococcaceae*, *Desulfovibrionaceae*↑; *S24-7*, *Bacteroidaceae*, *Paraprevotellaceae*, *Lactobacillaceae*, *Erysipelotrichaceae*↓; **Genus**: *Oscillospira*, *Ruminococcus*↑ *Bacteroides*, *Prevotella, Lactobacillus*↓ **Species**: R*uminococcus gnavus*↑; *Lactobacillus reuteri*↓	Abundance of lamina propria Th17, but not Th1 cells is highly correlated with the severity of arthritis. Elimination of gut microbiota reduces intestinal Th17 cells, decreases serum amyloid A expression in ileum and synovial tissues, and attenuates arthritis.	[14]
Pre-arthritic CIA susceptible mice VS Pre-arthritic CIA resistant mice	/	Animal study	16S rRNA (regions V3–V4)	**Phylum:** *Firmicutes*, *Bacteroidetes*, *Proteobacteria*↑ **Family:** *Lactobacillaceae*↑**;** *Desulfovibrionaceae*, *Lachnospiraceae*↓ **Genus:** *Lactobacillus*↑; *Enterorhabdus, Alistipes, Desulfovibrio*↓ **CIA post-onset:** **Family:** *Desulfovibrionaceae*, *Lachnospiraceae*↑; *Lactobacillaceae*↓	Elevated levels of serum IL-17 and Th17 cells in the spleen of CIA-sensitized mice. Microbiota colonization of CIA-sensitized mice increased serum IL-17 concentrations in GF mice, the ratio of splenic Th17 to Treg cells, and increased the incidence and severity of arthritis after type II collagen induction.	[35]
DRB1 0401 transgenic mice VS DRB1 0402 transgenic mice	/	Animal study	16S rRNA (regions V1–V3)	**Genus:** *Allobaculum*, *Clostridia*↑	Increased intestinal permeability and Th17 cell profile in DRB1 0401 mice.	[68]
Lewis rats before the onset of AIA VS Naïve Lewis rats	/	Animal study	16S rRNA (regions V3–V4)	**Class:** *Bacteroidia*, *Coriobacteriia*↓ **Genus:** *Clostridia*↑	Increased expression of intestinal inflammatory factors IL-8, IL-33, IL-17 mRNA and increased number of intestinal CD4+ and CD8+ T cells. Elevated plasma zonulin levels.	[18]

A reduction in *Bacteroides* represents another prominent feature of early RA [6,9], although its persistence in established disease remains uncertain [6,8]. Both *Prevotella* and *Bacteroides* appear closely linked to clinical responses to methotrexate (MTX). By applying Dirichlet Multinomial Mixture modeling, Jun et al. [10] identified two genus-level RA enterotypes, dominated respectively by *Prevotella* and *Bacteroides*. The *Prevotella*-rich enterotype showed a strong association with subsequent MTX responsiveness, possibly reflecting distinct biosynthetic or metabolic capacities of the underlying microbial communities.

*Collinsella*, a relatively rare *Actinobacteria* genus in the gut, has demonstrated the ability to increase intestinal permeability *in vitro* [11]. Elevated *Collinsella* abundance is frequently observed in individuals with chronic RA and is independently correlates with inflammatory activity [11,12]. Increased relative abundance of *Ruminococcus* is commonly seen in both early and established RA, correlating with disease activity and autoantibody levels [9,13,14], and has therefore been proposed as a potential microbial biomarker [13].

The gut microbiota is shaped by numerous host and environmental variables, and despite rigorous matching of participants for ethnicity, diet, sex, and age, many clinical cohorts have overlooked the confounding influences of body mass index and smoking *–* two major determinants of microbial composition that also affect RA risk. Uneven distribution of these factors may obscure or exaggerate true microbiome–RA associations if not properly accounted for. Future investigations must therefore statistically or experimentally minimize their confounding effects. Additional variables *–* including the duration of serum autoantibody positivity in PreRA individuals, disease activity and inflammatory status in NORA and RA, patients, and differences in pharmacologic exposure *–* may all influence the composition and abundance. The degree to which these parameters should be controlled warrants careful consideration in studies aimed at defining RA-associated microbial signatures. Current evidence draws largely from cross-sectional cohorts, which cannot clarify causal relationships between gut dysbiosis and RA pathogenesis. Longitudinal investigations are essential for defining dynamic microbial trajectories across disease stages and assessing how these shifts contribute to RA onset and progression.

## 3. By what means does the gut microbiota contribute to RA?

### 3.1. Impairment of intestinal permeability

On the one hand, damage to the intestinal mucosal barrier increases epithelial permeability, enabling non-self-antigens to translocate into the host and amplify systemic inflammation, destabilize self-tolerance, and ultimately disturb immune homeostasis. On the other hand, alterations in the gut microbiota further compromise barrier integrity and contribute to the initiation and progression of RA [15]. Elevated permeability also permits gut-resident immune cells to exit the intestine and initiate extraintestinal inflammatory responses [16,17]. Restoring tight–junction integrity has therefore emerged as a promising preventive strategy, as barrier disruption has been documented in both mice and humans prior to the onset of clinically detectable arthritis [6,16].

#### 3.1.1. Early stage of RA

Serum zonulin has been proposed as a biomarker for the transition from autoimmunity to inflammatory arthritis, as individuals with PreRA exhibit markedly elevated zonulin levels, and longitudinal analyses indicate that those with concentrations above 10 ng/mL during the preclinical phase face a substantially increased risk of developing RA within one year [16]. Collagen-induced arthritis (CIA) mice similarly show heightened serum zonulin and increased intestinal permeability before arthritis onset. This shift appears to be microbiota-driven, as transferring the CIA microbial community to germ-free (GF) mice induces comparable permeability changes [16]. Administration of a zonulin antagonist prior to CIA onset selectively restored epithelial integrity and attenuated subsequent arthritis severity [16]. Elevated zonulin levels before arthritis onset have also been reported in adjuvant-induced arthritis (AIA) rats [18]. Fecal microbiota transplantation (FMT) from PreRA individuals disrupts mucosal barrier integrity and increases permeabilit in recipient mice, and *in vitro* experiments confirm that gut bacteria from these PreRA individuals downregulate zonula occludens-1 (ZO-1) in Caco-2 cells at both transcriptional and protein levels [19].

#### 3.1.2. Established stage of RA

Patients with RA display decreased colonic ZO-1 expression and elevated serum and fecal zonulin levels, and persistently increased permeability may facilitate microbial translocation into the synovial fluid during stage IV RA [16,20,21]. The zonulin agonist AT-1002 exacerbates arthritis in experimental models, whereas treatment with the zonulin antagonist larazotide reduces osteoclast numbers and mitigates joint inflammation [16]. Hypoxia-inducible factor-2α (HIF2α) a mucosal epithelial transcription factor essential for maintaining intestinal integrity, has also been implicated in RA pathogenesis. Wen et al. [22] demonstrated that inhibiting intestinal HIF2α protects against CIA by preserving barrier function through selective suppression of the pore-forming protein CLDN15.

### 3.2. Modulation of the immune response between the ‘gut–joint axis’

Studies in GF mice highlight the critical role of the gut microbiota in immune maturation, including the development of gut-associated lymphoid tissue (GALT), which is central to the induction of mucosal tolerance to self-antigens [23]. Current evidence supports a model in which gut dysbiosis drives hyperactivation of innate and adaptive immune responses within the mucosa. Activated immune cells may then traverse the compromised epithelial barrier and enter systemic circulation, promoting widespread immune dysregulation. These cells, along with downstream inflammatory mediators, can subsequently infiltrate joints tissues, initiate synovitis, and ultimately promote cartilage and bone, destruction *–* events that culminate in the development of RA.

#### 3.2.1. Modulatory effects on immune cells

##### 3.2.1.1. T cell dysregulation in RA

**Figure 2.**
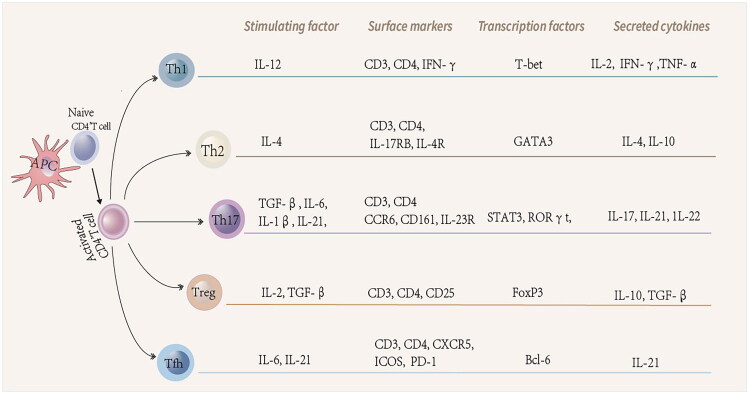
Differentiation of naive CD4+ T cells. Initial CD4+ T cells are activated and differentiated into various Th cell subpopulations in response to antigenic stimulation and cytokine signaling, and their differentiation process is dependent on the expression of specific transcription factors induced by specific cytokines.

T-cell function and the homeostatic balance among T-cell subsets are strongly shaped by the gut microbiota and its metabolites. Following antigenic stimulation together with cytokine cues, naïve CD4^+^ T cells become activated and differentiate into distinct helper T-cell (Th) lineages, a process orchestrated by cytokine-dependent induction of lineage-specific transcription factors (Figure 2) [24]. Key pro-inflammatory cytokines implicated in RA *–* interleukin-2 (IL-2), tumor necrosis factor-α (TNF-α), and IL-17 *–* are primarily produced by Th1 and Th17 cells, whereas Th2- and Treg-derived IL-4 and TGF-β exert protective, disease-limiting functions. The frequency of T follicular helper (Tfh) cells positively correlates with disease activity in RA patients [25], prompting substantial interest in their pathogenic contributions. Tfh cells support germinal-center (GC) B-cell differentiation into plasma cells and memory B cells; their depletion compromises antibody formation [26], while dysregulated Tfh activity enhances autoantibody production and accelerates autoimmune progression [27,28]. T follicular regulatory (Tfr) cells *–* recently identified as a specialized Treg subset *–* modulate GC responses [29,30]. These cells restrain Tfh activity by dampening the production of effector cytokines, including IL-4, interferon-γ, and IL-21, which are critical for B-cell activation [31,32].

##### 3.2.1.2. Early stage of RA

The gut microbiota undergoes notable alterations during the immunization phase of CIA in mice. Among intestinal lamina propria T-cell subsets, Th17 cells *–* but not Th1 cells *–* show a strong correlation with arthritis severity; depletion of the microbiota attenuates Th17-driven inflammatory processes [14]. Compared with mice receiving FMT from healthy human donors, mice colonized with PreRA microbiota display increased Th17 cell frequencies and elevated expression of associated inflammatory cytokines in mesenteric lymph nodes (MLNs) and Peyer’s patches (PPs), without corresponding changes in Th1 or Th2 frequencies. However, Th1, Th2, and Th17 frequencies in the spleen remain comparable to those in healthy-control FMT mice [19]. These findings indicate that microbial communities disrupt intestinal immune homeostasis and impose a pro-inflammatory Th17-cell bias at mucosal sites, thereby predisposing recipient mice to more severe arthritis than mice colonized with healthy microbiota [19]. Members of the *Bacteroides* genus, which degrade complex dietary carbohydrates to generate nutrients for the host, contribute to mucosal tolerance by promoting Treg-cell differentiation in the intestine [33]. NORA patients consistently show depletion of beneficial *Bacteroides* taxa [6]. Administration of *Bifidobacterium adolescentis* (*B. adolescentis*) before the initial immunization in CIA rats restores immune balance by expanding Treg populations in MLNs, suppressing TNF-α expression, and maintaining fecal short-chain fatty acid (SCFA) concentrations. These effects collectively confer both preventive and therapeutic benefits, in contrast to administration after immunization [34]. Additionally, microbiota from CIA-susceptible mice increases the splenic Th17/Treg ratio in GF mice and enhances both the incidence and severity of arthritis following type II collagen (CII) immunization [35].

Transgenic arthritic mouse models spontaneously develop joint inflammation that mirrors features of RA. In contrast to the findings of Fan et al. [34], *B. adolescentis* colonization in K/BxN mice selectively expands antigen-specific Th17 cells within the intestinal mucosa and exacerbates arthritis [36]. Whether this discrepancy reflects intrinsic differences among RA models remains unresolved. GF K/BxN mice are protected from arthritis due to an absence of IL-17–producing T cells. Colonization of GF K/BxN mice with *segmented filamentous bacteria* (*SFB*) restores lamina propria Th17 cells and induces autoantibody production, thereby triggering arthritis [37]. Teng et al. [38] further demonstrated that PP Tfh cells are essential for *SFB*-driven autoimmune arthritis in this model. Although autoantibodies are produced predominantly in systemic lymphoid tissues rather than in PPs, their work established that *SFB* aggravates disease by driving PP Tfh cell differentiation and promoting their migration to systemic sites, where they amplify circulating Tfh responses and autoantibody production [38]. Block’s findings [39] provided additional support by showing that K/BxN microbial communities continue to drive disease even in the absence of IL-17, whereas conditional deletion of Bcl6 in T cells abrogates Tfh differentiation and prevents arthritis. Maeda et al. [40] reported that transplantation of *P. copri*-dominated gut microbiota from NORA patients into zymosan-treated GF SKG mice leads to more severe arthritis than transplantation of microbiota from healthy controls or from RA patients lacking *Prevotella* enrichment. *Prevotella*-dominant microbial communities induce localized joint and intestinal autoreactive T-cell IL-17 responses directed against the RA-associated autoantigen RPL23A. In contrast, oral administration of *Prevotella histicola* (*P. histicola*) confers both preventive and therapeutic benefits in mouse arthritis models by modulating intestinal Th17/Treg balance and reducing epithelial permeability [17].

##### 3.2.1.3. Established stage of RA

Members of the *Ruminococcus* genus contribute to the degradation of resistant starch but have also been implicated in multiple inflammatory and immune-mediated disorders [41]. Consistent with observations by Sun et al. [9], Wu et al. [13] identified elevated *Ruminococcus* abundance in RA patients, positively correlating with disease activity and accompanied by reduced peripheral Tfr cell frequencies.

*Parabacteroides*, is widely regarded as beneficial to human health, shows a reduced abundance in RA patients; levels of *Parabacteroides distasonis* inversely correlate with DAS28 scores. Oral supplementation with *Parabacteroides distasonis* alleviates joint inflammation in CIA mice by restoring Th17/Treg balance in both the spleen and intestine [9]. *Lactobacillus casei* (*L. casei*) likewise mitigates arthritis in CIA rats by modulating Th17/Treg ratios and altering plasma metabolites [42]. Broad-spectrum antibiotic treatment partially depletes the gut microbiota in CIA mice, reducing lamina propria Th17 cells and ameliorating arthritis [14]. In contrast, oral administration of the periodontal pathogen *Porhyromonas gingivalis* promotes joint inflammation in CIA mice by increasing the proportion of Th17-cell in MLNs [43].

##### 3.2.1.4. Regulating B cells and antibodies

A diverse gut microbiota together with an intact mucosal barrier maintains GALT in a state of sustained yet controlled immune activation. This equilibrium stabilizes GC responses even under high antigenic pressure, thereby shaping how B cells recognize antigen and determine the specificity and quality of antibodies produced. The microbiome instructs dendritic cells (DCs) and follicular DCs to promote the differentiation of IgA-secreting effector B cells; in turn, secretory IgA feeds back to regulate microbial composition and function within the intestinal ecosystem [44]. Through coordinated engagement of both T-cell-dependent and T cell-independent pathways, microbial communities and their metabolites orchestrate multiclass B-cell responses that critically influence the balance between mucosal tolerance and autoimmunity [45–47].

##### 3.2.1.5. Early stage of RA

Animal studies have proposed a link between specific gut microbial taxa and the generation of PreRA-associated autoantibodies, raising the possibility that the intestinal mucosa may serve as a site of early antibody production [38,48–50]. Several clinical investigations have indirectly suggested the intestinal mucosa as a potential site of origin for ACPAs [51–53]. However, by detecting ACPAs, anti-carbamylated protein antibodies, and anti-acetylated protein antibodies in fecal samples and ileal lavage fluid from seropositive RA patients, Veerle et al. did not provide direct evidence that the intestinal mucosa is the source of these antibodies [54]. Current data suggest that the gut microbiota may contribute to systemic autoantibody production before clinical disease onset, although the precise anatomical origin of these antibodies remains to be conclusively determined.

##### 3.2.1.6. Established stage of RA

Associations between the gut microbiota and systemic B-cell responses persist into the established phase of RA. Chen et al. [12] demonstrated that reduced microbial species richness (α-diversity) correlates with elevated RF levels and disease progression. Experimental evidence further indicates that *Lactobacillus sakei* (*L. sakei*) administration increases serum IL-10 concentrations in CIA mice through enhanced differentiation of regulatory B cells (Bregs) rather than through Treg-mediated mechanisms [55].

##### 3.2.1.7. Regulating macrophage

Macrophages play a central role in inflammatory regulation and represent a critical link within the gut–joint axis of RA, particularly during established disease. Gut microbial signals and metabolites influence intestinal macrophage polarization, thereby modulating inflammatory intensity, while microbial antigens presented by macrophages activate CD4^+^ T cells and shape mucosal adaptive immunity [9,56]. In parallel, polarized macrophages or those carrying bacterial DNA may migrate from the gut to synovial tissues, where they contribute to joint inflammation [57,58]. Subclinical intestinal inflammation characterized by increased macrophage infiltration has also been documented in a subset of patients with early-stage RA [16].

Macrophage differentiation into osteoclasts constitutes a pivotal checkpoint linking inflammation to bone destruction in RA. Upon polarization toward an M1 phenotype, macrophages secrete IL-6 and IL-23, promoting Th17-cell differentiation and subsequent IL-17 production; IL-17 then accelerates osteoclastogenesis by further driving macrophage-to-osteoclast conversion [59]. Microbiota-mediated regulation of osteoclast differentiation has been well characterized, particularly in oral diseases. Certain probiotics, including *Bifidobacterium longum* subspecies modulate immune-cell differentiation and regulate IL-22 and IL-23 expression, thereby suppressing osteoclast formation and alleviating arthritis severity [60].

#### 3.2.2. Migration of immune cells: from intestinal mucosal immunity to systemic immunity

This section addresses the early stages of RA, during which accumulating evidence suggests that autoimmune responses may originate within GALT, spatially distant from the joints. Altered intestinal barrier integrity and increasedepithelial permeability may facilitate the dissemination of intestinal immune cells to synovial sites. Using KikGR mice to track cellular migration, Takahashi [49] demonstrated that both Tfh cells and B cells migrate from the colonic PPs to the draining lymph nodes (DLNs) following immunization. These findings identify distal GALT as a primary site for autoimmune initiation in the CIA model and highlight the role of the gut microbiota in providing diverse adjuvant signals that support this process. Teng et al. [38] also showed that SFB- induced arthritis depends on the migration of Tfh cells from PPs to peripheral lymphoid tissues. In the K/BxN mouse model, a population of Th17 cells expressing the intestinal homing integrin α4β7 persists within the spleen. Modulation of α4β7 expression on Tfh cells alters their trafficking between the intestinal mucosa and systemic inflammatory sites, resulting in either exacerbation or attenuation of joint inflammation [61]. An alternative explanation for the presence of α4β7-expressing Th17 cells in the spleen has been proposed by Marietta et al. [17], who identified CD103^+^ DCs originating from the intestinal lamina propria within the spleens of CIA mice. These DCs may migrate from the intestine and locally induce α4β7^+^ Th17-cell differentiation.

Direct evidence for gut-to-joint immune cell trafficking in human RA remains limited. However, studies in related conditions, such as spondyloarthritis, documented identical T-cell clones in both intestinal and joint tissues several decades ago [62]. Further support comes from observations that human intestinal immunoblasts bind efficiently to synovial high endothelial venules, while exhibiting minimal adhesion to those of peripheral lymph nodes [63,64]. These findings lend indirect support to the concept that intestinal mucosal immune cells can access joint tissues in inflammatory arthritis. On the basis of emerging evidence for immune-cell trafficking from mucosal to systemic compartments, therapeutic strategies aimed at disrupting this migratory axis may represent a promising avenue for arthritis intervention.

### 3.3. Genetic risk and molecular mimicry

#### 3.3.1. Genetic risk

RA is a multifactorial autoimmune disorder in which both genetic predisposition and environmental exposures contribute to disease development. Genome-wide association studies have identified numerous susceptibility loci involved in T-cell differentiation, peptide binding affinity, and antigen selection, including *HLA-DRB1*, *TNFAIP3*, *PTPN22*, and *PADI4* [65]. Among these, *HLA-DRB1* shows the strongest association with autoantibody-positive RA, with more than 70% of ACPA-positive patients carrying this allele [66]. Environmental factors are thought to act as critical triggers in genetically susceptible individuals, and marked alterations in the gut microbiota have been reported even in clinically healthy carriers of the *HLA-DRB1* risk allele [67]. Studies using *HLA-DR* transgenic mice further demonstrate that gut microbial composition is, at least in part, shaped by host *HLA* genotype [68]. This bidirectional interaction between intestinal microbiota and RA risk alleles exemplifies the combined influence of genetic and environmental factors during the early stage of RA.

Human cohort studies provide additional support for this gene–microbiota interplay. Wells et al. [69] stratified *Prevotella* taxa by predicted species using SILVA-based nomenclature and observed a significant association between RA genetic risk and gut microbiota composition in individuals without clinically apparent disease. The strongest association involved *Prevotella_7* (species undetermined), which positively correlated with RA risk genotypes. Although positive log-fold changes were also observed for *Ruminococcaceae_UCG-14, Rikenella*, and *Shigella*, these associations were substantially weaker than that observed for *Prevotella_7* [69]. In contrast, Scher et al. [6] reported that NORA patients exhibited an expansion of *P. copri*, with its relative abundance inversely correlated with specific *HLA-DRB1* alleles. Based on these findings, the authors proposed that individuals lacking genetic susceptibility may require a higher *P. copri* burden to initiate disease, whereas carriers of risk alleles reach a pathogenic threshold at lower microbial abundance. Discrepancies between these studies may also be associated with demographic differences or other confounding factors. Beyond risk alleles themselves, environmental influences on genetic susceptibility may also be mediated through molecular mimicry by gut microbes.

#### 3.3.2. Molecular mimicry

Molecular mimicry refers to the structural homology between pathogen-derived antigens and host autopeptides, leading to cross-reactive activation of autoreactive T or B cells and subsequent autoimmunity. Gut microbes can engage the immune system and elicit T-cell responses to self-antigens through molecular mimicry [70].

##### 3.3.3. Early stage of RA

Maeda et al. [40] demonstrated that naïve CD4+ T cells isolated from SKG mouse spleens, when co-cultured with *P. copri*-stimulated DCs, produced high levels of IL-17 in response to the RA autoantigen RPL23A. Subsequently, Pianta et al. [71] identified *HLA-DR*-presented peptides from the 27-kd protein of *P. copri* (Pc-p27) capable of inducing Th1 responses in 42% of NORA patients. They also found that both NORA and chronic RA exhibited either an IgA-like antibody response to Pc-p27 or to *P. copri*, or developed IgG *P. copri* antibodies [71].

##### 3.3.4. Established stage of RA

In more than half of patients with RA, two autoantigens *– N*-acetylglucosamine 6-sulfatase (GNS) and filamin A (FLNA) *–* have been identified as shared targets of T- and B-cell immune responses [72]. *HLA-DR*–presented peptides derived from GNS exhibit sequence homology with epitopes from *Prevotella* and *Parabobacteroides* species, whereas *HLA-DR*–restricted FLNA peptides share homology with protein epitopes from *Prevotella* and *Butyricimonas* species [72]. These findings further strengthen the mechanistic link between intestinal mucosal immunity and joint-directed autoimmune responses in RA. Zhang et al. [73] additionally demonstrated that microbial genes from *Clostridium*, *Eggerthella*, *Bacteroides,* and *Citrobacter* in RA patients display molecular mimicry with RA-associated antigens, including collagen XI and HLA-DRB1*0401, and many genes belonging to metagenomic junction group were enriched in RA intestinal samples. CII, a principal structural component of articular cartilage and a commonly used autoantigen in experimental arthritis models, elicits pathogenic autoantibodies detectable in the serum and synovial fluid of RA patients [74]. Peptides derived from *Candida albicans* and *Streptococcus sanguinis* cross-react with CII-specific immune cells and modulate CII-induced arthritis in mice [75,76]. Beyond molecular mimicry, bacterial invasion has been proposed to induce host cell necrosis and apoptosis, leading to extracellular exposure of intracellular autoantigens that subsequently activate B cells and promote autoantibody production [77].

### 3.4. Gut microbes influence RA through derived metabolites

The gut–joint axis in RA is also profoundly shaped by microbial metabolites [8,78], and both gut dysbiosis and metabolic perturbations have been documented in individuals at elevated risk for disease development [19]. In addition to extensively studied SCFAs, other microbial-derived metabolites *–* including amino acids and bile acids *–* are significantly altered in RA patients, although their mechanistic contributions remain incompletely understood.

#### 3.4.1. SCFAs

SCFAs, produced through bacterial fermentation of dietary fibers, include acetate, propionate, isobutyrate, butyrate, isovalerate, valerate, and caproate. Reduced fecal concentration of acetate, propionate, butyrate, and valerate have been reported in RA patients [78], with subsequent studies confirming decreased butyrate and propionate levels in individuals with NORA and inactive disease [49,79]. In contrast, findings regarding circulating SCFA concentrations remain inconsistent across studies [79,80].

SCFAs exert immunomodulatory effects through epigenetic mechanisms, including regulation of histone acetyltransferase and deacetylase activity; and them, butyrate functions as the most potent histone deacetylase inhibitor [81,82]. Through these pathways, SCFAs suppress CD4^+^ T-cell activation while promoting the expansion of IL-10–producing regulatory B (B10) cells, thereby preserving immune equilibrium [82–84]. In contrast to long-chain fatty acids, SCFAs bypass carnitine palmitoyltransferase 1 and directly enter mitochondria, where they are efficiently oxidized to generate cellular energy [85]. Consequently, the metabolic features of RA T cells *–* including mitochondrial dysfunction and reduced ATP production *–* may be partially attributable to a reduced level of SCFAs [86].

Given that the gut microbiota in RA is marked by depletion of butyrate–producing bacteria alongside expansion of butyrate consuming taxa [80,87], the role of butyrate in RA pathogenesis has attracted particular attention (Table 2). Although fecal acetate and propionate levels are likewise reduced in RA patients, whether augmenting luminal concentrations of these metabolites confers protection against disease onset or progression remains uncertain [49,78,88]. Accordingly, this section focuses primarily on the mechanistic actions of butyrate in the context of RA.

**Table 2.** Mechanism of action of butyrate in RA.

Country	Human subjects and results	Animal model	Mode of administration and dosage	Key findings	Reference
China	RA(9) VS HC(10) Acetate, propionate, and butyrate↓	CIA DBA/1J mice	The drinking water of mice was supplemented with the three SCFAs (acetate, propionate, and butyrate; all 150 mM)	Mice intestinal luminal three SCFAs concentrations↑. All three SCFAs promote Bregs differentiation and reduce migratory B cells and follicular B cells frequency, effectively controlling the initial phase of the autoimmune response rather than the effector phase of arthritis development.	[78]
China	NORA(40) VS HC(29) Butyrate↓	DBA/1 mice	Diet containing 20% butyrate	Butyrate can balance Tfh cells and Treg cells in DLN and PP, reduce serum antibodies, and improve arthritis in mice.	[80]
Japan	NORA(31) VS HC(41) Acetate, propionate, and butyrate↓	CIA and CAIA DBA/ 1 J mice	Low-fiber diets with HAMSBs maintained the three SCFAs (acetate, propionate, and butyrate) at physiological concentrations	Mice intestinal luminal three SCFAs concentrations↑. Acetate, propionate: do not have a significant effect on arthritis. Butyrate: Tfr cell differentiation↑, Tfh cell differentiation↓, autophagy antibody IgG2a↓. Butyrate reduces the production of autoantibodies rather than immune complex induction to alleviate the development of arthritis; Restrict the development of GC during CIA initiation to inhibit the production of CII-specific IgG.	[49]
England	RA(19) VS HC(20) Butyrate and propionate↓; no difference in acetate	AIA DBA/1 mice	The drinking water of mice was supplemented with the three SCFAs (acetate, propionate, and butyrate; all 150 mM)	Acetate, propionate: do not have a significant effect on arthritis. Butyrate: Bregs differentiation↑, differentiation of GC-B cells and plasma blast cells↓, arthritis symptom improvement.	[79]
/	/	CIA DBA/1J mice	Intraperitoneal injection of 100 mg/kg butyrate	TH17/Treg ratio in DLN↓, arthritis improvement.	[90]
/	/	AIA C57BL/6 Mice	The drinking water of mice was supplemented with the three SCFAs (acetate, propionate, and butyrate; all 300 mM)	Acetate, propionate: do not have a significant effect on arthritis. Butyrate: inhibits cytokine production (e.g., IFN-g, IL-4) by iNKT cells, thereby attenuating AIA.	[149]
/	/	CIA DBA/1J mice	The drinking water of mice was supplemented with butyrate (100 mM)	Butyrate promotes Treg cell differentiation, and Treg cells produce the anti-inflammatory cytokine IL-10, which further inhibits the production of Th17 cells and ameliorates arthritis.	[89]

##### 3.4.2. Early stage of RA

In the CIA model, administration of butyrate before disease onset prevents both initiation and progression of arthritis by promoting Tfr-cell differentiation and dampening the CII-driven GC responses [49]; In contrast butyrate fails to inhibit collagen antibody-induced arthritis (CAIA) when administered after booster immunization [49], indicating that its protective effects primarily target autoantibody generation rather than immune complex–mediated inflammation. In addition, Takahashi et al. [49] reported that CIA mice lose resistance to arthritis when butyrate is administered after booster immunization, indicating that butyrate suppresses CII-specific IgG production by constraining GC development during the immune-priming phase. The superior efficacy of butyrate when delivered prior to, rather than after, disease onset has also been reported by Yao et al. [78].

##### 3.4.3. Established stage of RA

Although *in vitro* experiments suggest that butyrate exerts minimal direct effect on Th17 cells [89], administration of high-dose butyrate during established disease suppresses osteoclast differentiation and alleviates arthritis by expanding Treg cells and restoring the intestinal and systemic Treg/Th17 balance [90,91]. By contrast, physiological concentrations of butyrate appear insufficient to sustain this balance or to prevent CAIA development [49].

Beyond its effects on T cells, butyrate also regulates B-cell responses. In antigen-induced arthritis models, butyrate activates aryl hydrocarbon receptor (AhR)-dependent transcriptional programs in B cells, thereby promoting differentiation of regulatory B-cells (Bregs) and suppressing the generation of GC B cells and plasmablasts [79]. Yao et al. [78] further demonstrated that multiple SCFAs *–* including acetate, propionate, and butyrate *–* enhance Breg differentiation, reduce the abundance of migratory and follicular B cells, and ameliorate arthritis *via* the free fatty acid receptor 2. The absence of disease suppression by butyrate in B-cell-deficient mice highlights the requirement for Bregs in mediating its anti-arthritic effects [79].

#### 3.4.4. Other organic acids

A retrospective Sweden study revealed that individuals who subsequently developed ACPA-positive RA exhibited elevated levels of lysophospholipids and tryptophan metabolites years before symptom onset than those without a diagnosis of RA [92]. Kyoto Encyclopedia of Genes and Genomes (KEGG) pathway enrichment analyses similarly identified increased amino acid and lipid metabolism in PreRA individuals compared with healthy controls [19], whereas amino acid biosynthesis pathways were depleted in NORA patients [8]. Comparable metabolic signatures have been observed in established RA, with amino acid levels normalizing following antirheumatic therapy [93,94]. Complementing these clinical findings, Seymour et al. [95] demonstrated in animal models that indole *–* a bacterial tryptophan metabolite produced by taxa such as *Parabacteroides copri*, *Collinsella*, and *Ruminococcus –* is required for CIA development by enhancing Th17 responses in both intestinal and systemic immune compartments.

Succinate, another microbially influenced metabolite, is markedly elevated in the synovial fluid of RA patients. In CIA mice, *P. copri* colonization combined with a high-fiber diet (HFD) results in excessive succinate accumulation [96]. Experimental studies have shown that succinate supplementation promotes macrophage pro-inflammatory polarization, increases Th17-cell frequencies, induces joint angiogenesis, and exacerbates synovial inflammation [96–98].

Bile acids represent an additional class of microbial metabolites with immunoregulatory functions. In RA animal models, intestinal bile acid levels are reduced, coinciding with depletion of bile acid–producing bacteria and an expansion of bile acid-consuming taxa [99,100]. *Bifidobacteria* and *Lactobacilli* constitute major the bile acid-metabolizing bacteria; through bile salt hydrolase–mediated deconjugation of glycochenodeoxycholic acid and glycocholic acids, these microbes contribute to bile acid–dependent immunomodulation and therapeutic attenuation of arthritis [100].

## 4. The application and prospect of regulating the gut microbiota in the treatment of RA

**Figure 3.**
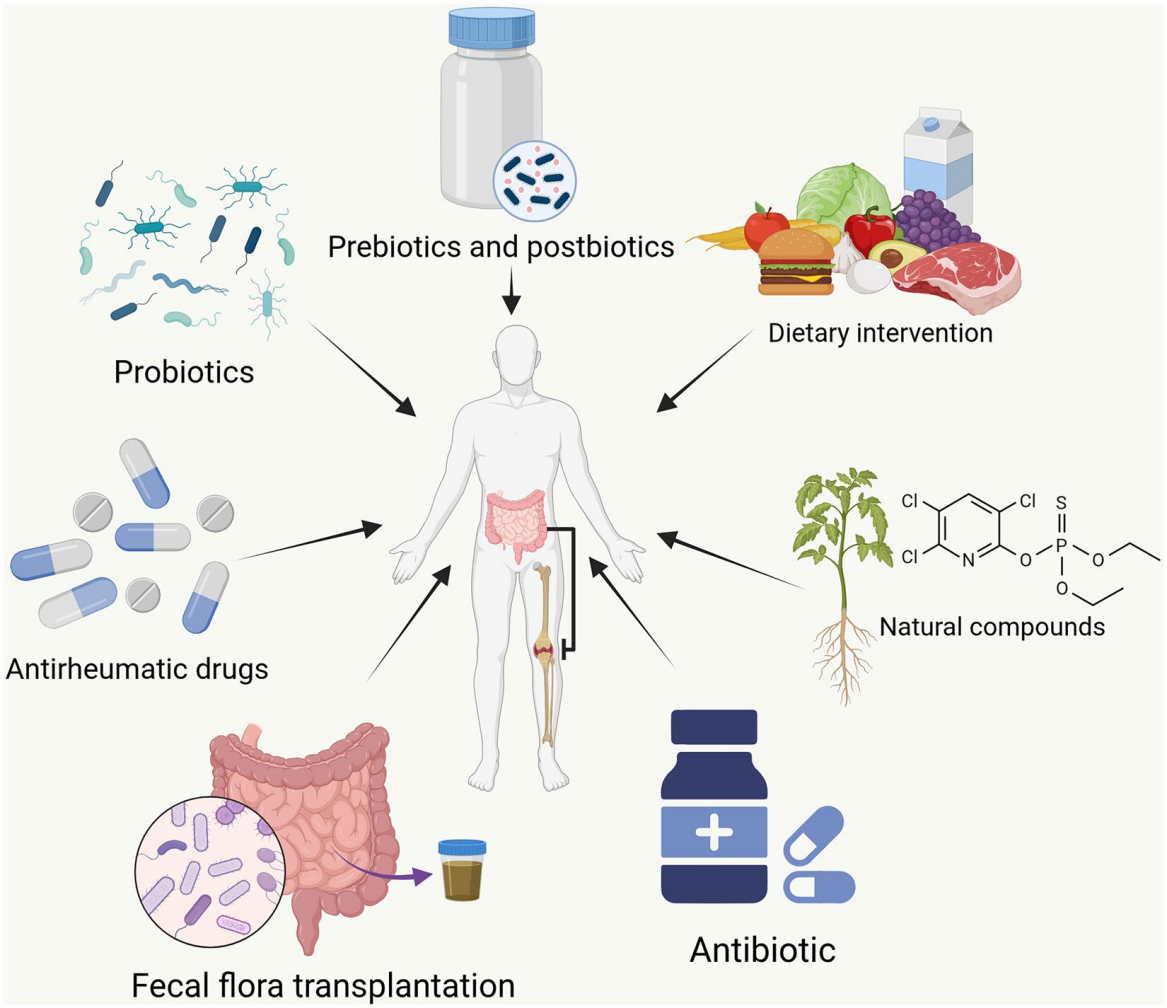
Potential strategies for gut–microbiota-based intervention in individuals with RA.

Although current therapeutic strategies have substantially improved symptom control and disease management in RA, they have failed to reverse the associated reduction in life expectancy. Most available treatments primarily target inflammatory pathways to alleviate pain and suppress disease activity, yet exert limited influence on the underlying pathogenic mechanisms of RA. As a result, lifelong treatment is required for the majority of patients. Notably, 30–50% of individuals ultimately discontinue or switch therapies due to serious adverse effects [101,102]. These limitations highlight the urgent need for safer and more effective interventions that directly target disease pathogenesis. This section summarizes emerging therapeutic and adjunctive strategies aimed at modulating the gut microbiota as a novel approach for RA management (Figure 3).

### 4.1. Probiotics

Clinical studies have reported beneficial effects of probiotic supplementation in RA. Patients receiving an 8-week course of probiotic capsules containing *L. acidophilus*, *L. casei*, and *B. bifidum* exhibited significant reductions in disease activity scores and serum high-sensitivity C-reactive protein levels compared with placebo controls [103]. Consistent findings were reported in randomized controlled trials conducted by Vaghef-Mehrabany et al. [104] and Alipour et al. [105], in which eight consecutive weeks of *L. casei* supplementation l ed to measurable improvements in clinical indices and inflammatory biomarkers. However, a 2018 meta-analysis incorporating four randomized controlled trials concluded that probiotic supplementation did not confer significant benefits over placebo in RA [106], highlighting ongoing uncertainty regarding clinical efficacy.

Preclinical studies provide stronger evidence for both therapeutic and preventive effects of probiotics. In PreRA mouse models, commonly used strains such as *B. adolescentis* and *Bifidobacterium longum* RAPO increase regulatory T-cell frequencies, restore immune equilibrium, and reduce arthritis incidence [34,107]. Other taxa, including *Lactobacillus helveticus* [108,109] and *Parabacteroides histicola* [17,110], effectively prevent arthritis development by modulating T-helper cell polarization and B-cell responses at mucosal and systemic levels. Notably, probiotic administration after disease onset often fails to confer protective effects [34,111].

Despite robust mechanistic support from experimental models, translation of probiotic efficacy to clinical settings remains inconsistent. This discrepancy likely reflects interspecies differences in gut microbiota composition, inherent limitations of animal models in recapitulating human RA, and substantial heterogeneity across clinical studies with respect to probiotic strains, patient characteristics, dosing strategies, timing of intervention, treatment duration, and concomitant therapies. Comprehensive stratified analyses based on a strain–host–context framework remain challenging due to the limited availability of comparable datasets. Nevertheless, existing evidence supports continued investigation into microbiota-targeted approaches. Identification of optimal probiotic strains *–* either alone or in rational combinations *–* and precise definition of target populations represent promising avenues for future research. In particular, findings from animal studies suggest that probiotic interventions applied during the preclinical or early disease phases may hold significant preventive potential.

### 4.2. Prebiotics and postbiotics

Prebiotics: Inulin, a widely used prebiotic, increases the abundance of beneficial gut bacteria through microbial fermentation. Dietary inulin supplementation administered prior to RA induction significantly suppresses joint swelling and attenuates arthritis development in experimental models [112]. Resistant starch, derived primarily from retrograded amylose, undergoes colonic fermentation following oral intake and promotes the production of SCFAs, including acetate and butyrate. Provision of a resistant starch–enriched HFD effectively prevents CIA in mice [88]. Lactoferrin, a bioactive glycoprotein abundant in milk and other secretions, exhibits broad antibacterial and antiviral properties. Oral lactoferrin administration markedly inhibits arthritis development in SKG mice [113]. Spirulina, a nutrient-dense cyanobacterium rich in essential minerals, vitamins, and micronutrients, is commonly used as a dietary supplement and exerts regulatory effects on intestinal dysbiosis. Treatment with Spirulina alleviates arthritic symptoms in AIA rats and reduces circulating levels of inflammatory mediators such as TNF-α and IL-6 [114].

Postbiotics: Postbiotics encompass inactivated microbial cells and bioactive metabolites derived from probiotic fermentation. Heat-killed *Propionibacterium freudenreichii MJ2* confers joint protection in CIA mice by suppressing osteoclast differentiation and limiting bone loss and erosion [115]. Similarly, inactivated *Lactiplantibacillus plantarum* cells and their culture supernatants ameliorate joint damage in RA models, reduce serum autoantibody concentrations, and decrease pro-inflammatory cytokine levels [116].

### 4.3. Dietary interventions

Diet represents a major determinant of gut microbial composition and functional capacity, and accumulating evidence supports dietary modulation as a viable strategy to delay disease onset and mitigate RA progression.

Mediterranean diet (MED): The MED emphasizes olive oil as the principal fat source, high consumption of vegetables and legumes, moderate intake of wine, and white meat (fish and poultry), and reduced sodium intake. This fiber-rich dietary pattern exerts antioxidant and immunomodulatory effects [117]. Clinical studies demonstrate that adherence to the MED reduces disease activity in RA patients [118], while long-term compliance induces sustained alterations in gut microbiota composition [119,120].

HFD: Vegetarian and plant-based diets, which are similarly rich in dietary fiber, have also been associated with improvements in RA disease activity [121], suggesting that fiber intake represents a key contributor to the benefits observed with both vegan and MED patterns. Building on this concept, Julian et al. [122] conducted a 28-day feasibility study of high-fiber supplementation in 36 RA patients and reported improvements in patient-reported outcomes, accompanied by reduced serum zonulin levels, increased circulating Treg frequencies, altered Th1/Th17 ratios, and decreased markers of bone erosion. High-fiber supplementation has additionally been shown to modify the intestinal *Firmicutes*-to-*Bacteroidetes* ratio in parallel with clinical improvement in RA patients [123]. In experimental settings, the resistant starch–enriched HFDs reverse the expansion of *Lactobacillus* and *Lachnoclostridium* genera, promote *Bacteroidetes* dominance, and attenuate joint inflammation in CIA mice [88]. Dietary fiber also serves as a primary substrate for microbial SCFA production, and SCFAs have demonstrated therapeutic efficacy in multiple RA animal models.

Fish oil: Fish oil is a rich source of omega-3 polyunsaturated fatty acids, and supplementation has been shown to significantly alleviate joint pain and reduce the duration of morning stiffness in patients with RA [124]. FMT experiments further demonstrated that the protective effects of tuna oil in CIA mice are mediated through the gut microbiota. Dietary tuna oil intervention restores microbial dysbiosis by increasing the abundance of *Bacteroidales*, *Clostridiales*, *Lactobacillales*, and *Desulfovibrionales* [125].

High-magnesium diet: Magnesium, the second most abundant intracellular cation, plays a critical role in numerous cellular biochemical processes. Laragione et al. [126] demonstrated that increasing dietary magnesium intake in RA model mice significantly suppresses the expression of pathogenic mediators, leading to reduced inflammation and attenuation of arthritis severity and joint damage. Subsequent antibiotic depletion and FMT experiments revealed that the beneficial effects of a high-magnesium diet are mediated by gut microbiome remodeling, characterized by reduced *Prevotella* abundance and enrichment of *Bacteroides* and SCFA–producing bacteria [126].

Other dietary therapies: In contrast to a high-magnesium diet, a high-natrium diet doubled the risk of RA in smokers [127] and is associated with increased cardiovascular adverse events in RA patients [128]. An elemental diet is a diet based on nutritional values such as amino acids, vitamins and simple sugars. A small study comparing two weeks of oral elemental diet with two weeks of oral prednisolone found that both were equally effective in disease management [129]. A high-quality cohort study found an inverse association between alcohol intake and RA risk [130], and the preventive effect of alcohol in experimental arthritis [131] suggested that alcohol may have a protective effect against RA.

### 4.4. Natural compounds

Studies of natural compounds derived from traditional herbs in RA therapy have also revealed the mechanism of gut microbes in this context. The gut microbiota may be a target for these high-molecular-weight, low-bioavailability natural compounds in the treatment of chronic diseases. Total glucosides of Paeony (TGP), a standardized extract from the root of *Paeonia lactiflora* Pall., are widely used as an immunomodulatory agent in the clinical management of RA. Animal studies have shown that, concomitant with arthritis amelioration, TGP reverses the gut–microbial taxonomic deviations observed in CIA rats compared with controls, increases the relative abundance of beneficial commensal flora (e.g. *Prevotellaceae*, *unclassified_Ruminococcaceae*, *Tenericutes*), modulates intestinal mucosal immunity [132], restores the mucosal barrier, and may modulate purine metabolism [133].

Berberine (BBR), an isoquinoline alkaloid and regulator of intestinal flora, has been used for decades to treat gastrointestinal disorders. Yue et al. [134] demonstrated that BBR attenuated CIA in a gut flora-dependent manner. Specifically, BBR reduced intestinal *Prevotella* abundance, increased butyrate-producing bacteria, suppressed nitrate generation, and maintained physiologic hypoxia of the mucosa, thereby exerting its therapeutic effect [134].

Sinomenine is a potent immunosuppressive and anti-inflammatory agent widely used for RA clinical treatment. Animal studies have shown that its therapeutic efficacy is largely attributed to AhR activation through specific modulation of *Lactobacillus* and microbial tryptophan metabolites [135].

Clematis triterpenoid saponins (CTSs), isolated from *Clematis manshurica* Rupr., significantly ameliorate arthritis in CIA rats by restoring the ratio of Gram-positive to Gram-negative bacteria but have no effect on the microbiota in healthy rats, suggesting that CTSs act as microbiota-balancing agents rather than alter specific bacterial taxa [136].

The therapeutic efficacy of numerous natural compounds in RA is well established. However, their use as preventive interventions remains largely unexplored, because potential adverse effects in healthy subjects or preclinical models have yet to be determined. Moreover, optimizing dose and timing for prophylactic administration presents significant challenges.

### 4.5. Antibiotic treatment

Antibiotic treatment can cause drastic changes in gut microbial diversity and community composition. Although clinical studies have linked antibiotic combination therapy to significant improvements in RA disease activity [137–139], prophylactic or excessive antibiotic use induces gut microbiota dysbiosis and is associated with an elevated risk of RA [140,141]. Thus, antibiotics can ameliorate established RA while potentially precipitating de novo RA in healthy individuals, implying that gut microbiota changes mediate this stage-dependent, bidirectional influence on disease.

### 4.6. Fecal flora transplantation

Although basic research has demonstrated that FMT can reconstitute the intestinal ecosystem and modulate innate and adaptive immune responses to exert therapeutic effects, clinical trials remain in their infancy. Zeng et al. [142] first reported successful treatment of a patient with 5-year refractory RA *via* FMT, and achieved a beneficial clinical response. Although this case indicates that FMT may be a treatment for RA, its clinical implementation remains fraught with challenges. First, FMT carries a risk of infectious adverse events; thus, ensuring participant safety is paramount. Second, the durability of microbiota changes induced by FMT has yet to be determined. Third, patient acceptance and adherence are essential for post-procedure monitoring of safety and efficacy, yet current acceptance rates are suboptimal. Consequently, the clinical translation of FMT for RA is still in its infancy. Rigorous donor–recipient screening, standardized protocols, and comprehensive education are all imperative before large-scale trials and routine use can be realized.

### 4.7. Antirheumatic drugs

A meta-analysis by Wang et al. [87] reported a significant reduction in gut microbial α-diversity in RA patients, a finding was predominantly observed in cohorts not receiving antirheumatic therapy. This observation suggests that treatment may partially restore microbial diversity in RA. Human gut microbes and their enzymatic activities influence drug bioavailability, therapeutic efficacy, and toxicity through both direct and indirect mechanisms, contributing to inter-individual variability in treatment responses. The gut microbiota has long been recognized as a key determinant of antirheumatic drug biotransformation, particularly for MTX, whose clinical efficacy and bioavailability a vary widely among patients. Numerous cohort studies have explored associations between MTX responsiveness and clinical parameters such as sex, age, disease activity, and glutamate concentrations, yet consistent predictors have not emerged. When conventional clinical variables fail to explain this variability, differences in gut microbiota composition and metabolic capacity have been proposed as a primary contributing factor. Artacho et al. [143] stratified NORA patients into MTX responders and non-responders and demonstrated *–* through metagenomic profiling and *ex vivo* MTX metabolism assays *–* that gut microbiota composition directly modulates MTX bioavailability and therapeutic efficacy [143]. The presence of distinct enterotypes between responders and non-responders was further supported by Qiao et al. [10], although the dominant microbial taxa identified differed from those reported in Isaac’s study. Geographic, environmental, and ethnic heterogeneity likely underlies these discrepancies. These findings indicate that development of microbiota-based predictive models for drug response will require not only larger, well-characterized clinical cohorts but also rigorous consideration of confounding variables. Such efforts are essential for advancing microbiome-informed precision medicine strategies in RA.

## 5. Window of opportunity for RA treatment

Traditional therapy for RA is initiated only after clinical onset, and prolonged treatment leads to cumulative adverse drug events. Therefore, a critical period likely exists during which the disease is more amenable to intervention; this phase is termed the ‘window of opportunity’ for RA. In a systematic literature review, Burgers et al. [144] identified an ‘old definition’ of the window of opportunity as the first two years after diagnosis, during which disease-modifying therapy is most likely to prevent severe joint damage and disability; substantial evidence has since supported this concept. The window of opportunity has since evolved with respect to both timing and therapeutic endpoints. The ‘new definition’ posits that this period may precede the fulfillment of classification criteria, during which intervention could prevent progression to classifiable RA; however, this concept still lacks randomized controlled trial support [144].

As mentioned above, certain ‘gut–joint axis’ targeting interventions are efficacious only if administered before immune tolerance is lost and arthritis becomes clinically evident; thus, therapeutic targeting of the gut microbiota and the mucosal barrier may constitute a breakthrough. If diet, probiotics, or natural compounds can rebalance immunity by correcting microbial dysbiosis in PreRA individuals, RA onset may be delayed or prevented. These hypotheses require comprehensive preclinical and long-term clinical studies to identify optimal formulations and verify preventive efficacy. Even more challenging, preventive intervention in asymptomatic pre-RA individuals raises formidable ethical issues, including the adequacy of conventional review mechanisms, the risk of overtreatment, an uncertain benefit–harm ratio, the extent to which asymptomatic participants can comprehend information about ‘possible future disease’ and ‘preventive intervention’, and liability allocation should the intervention fail or produce adverse outcomes.

## 6. Discussion

To date, gut microbiota research in RA has focused predominantly on bacterial communities, with comparatively few studies examining the roles of fungi or helminths [145,146]. Substantial gaps therefore remain in understanding the contribution of non-bacterial microorganisms to RA pathogenesis. As outlined above, the gut microbiota profoundly shapes the intestinal microenvironment and epithelial homeostasis, thereby constituting a critical interface through which environmental risk factors influence RA development. Despite this recognition, current investigations into microbiota–disease relationships remain constrained by several conceptual and methodological limitations. First, inter-individual variability in gut microbial composition is often substantial and difficult to control, at times exceeding disease-associated effects and complicating the identification of reproducible microbiome signatures. Second, attributing unexplained pathogenic mechanisms broadly to the ‘microbiota’ risks oversimplification. As emphasized by Brüssow [147], microbiome research has become encumbered by conceptual and terminological and ambiguities, particularly regarding the absence of a clear and consensual definition of ‘ecological dissonance,’ which may obscure mechanistic interpretation. Third, most studies relying on 16S rRNA gene sequencing achieve taxonomic resolution only at the genus level, limiting insight into strain-specificity effects that may be critical for understanding functional consequences. Careful consideration of strain-level variation is therefore essential when interpreting observed microbial differences between groups. Finally, although feces sampling offers practical advantages, fecal microbiota do not fully capture the spatial heterogeneity of the intestinal ecosystem. A priori evaluation is required to determine whether fecal-based analyses adequately address the biological questions under investigation.

Overall, it is essential to account for and control inter-individual confounders when analyzing gut microbiome composition and to recognise variations attributable to ethnicity, geography, and diet, which are not caused by disease.

Beyond descriptive profiling of community composition, hypothesis-driven investigation of specific microbial taxa may offer greater mechanistic clarity. As discussed previously, distinct species *–* and even strains *–* within genera such as *Lactobacillus* and *Prevotella* exert different effects on RA pathogenesis and disease activity, indicating that immunomodulatory functions of the gut microbiota are frequently species- and strain-dependent. Genus-level classifications can obscure these biologically meaningful differences; refinement toward strain-specific functional traits will be essential for developing reproducible precision–probiotic strategies and personalized dietary interventions. Although microbial community structures vary widely among individuals, metabolic outputs appear comparatively conserved, suggesting that different taxa may converge on shared pro-inflammatory or anti-inflammatory pathways at local or systemic levels. Future research should therefore extend beyond cataloging compositional changes to focus on alterations in the abundance and function of defined microbial groups *–* or even individual strains *–* alongside shifts in the metabolome and their effects on immune tolerance. Particular emphasis should be placed PreRA, where microbiota-targeted interventions may hold greater preventive potential than treatments initiated after disease establishment. At present, however, RA prevention research remains largely confined to animal models. Translation to clinical trials poses significant challenges, including identification of appropriate target populations, determination of optimal intervention windows, logistical barriers to recruitment, and unresolved ethical considerations.

## 7. Conclusion

Overall, gut–microbiota dysbiosis contributes to the onset and progression of RA *via* multiple pathways. RA follows a prolonged disease course, during which the gut microbiota is influenced by numerous environmental and host factors. Unlike previous reviews, in this review, we herein define the interval from loss of immune tolerance to tissue inflammation as the early phase of RA, and the transition from acute to chronic, persistent synovitis as the established phase. This staged discussion provides a longitudinal framework essential for understanding the role of the gut microbiota in RA. Moreover, targeting the gut microbiota appears to hold greater preventive than therapeutic potential for RA, offering a more promising strategy before clinical disease onset. It must be emphasized that mechanistic conclusions derived from animal models *–* including the causal roles of specific taxa and the efficacy of microbiota-targeted interventions *–* remain to be adequately validated in human RA. Likewise, although FMT in these models has provided proof-of-concept evidence for underlying mechanisms, supportive data from human studies are still lacking. These translational limitations must be carefully considered when extrapolating laboratory findings to clinical practice.

## Compliance with ethics guidelines

This article is based on previously conducted studies and does not contain any studies with human participants or animals performed by any of the authors.

## Data Availability

Data sharing is not applicable to this article as no data were created or analysed in this study.
